# pH-responsive mesoporous silica nanoparticles employed in controlled drug delivery systems for cancer treatment

**DOI:** 10.7497/j.issn.2095-3941.2014.01.003

**Published:** 2014-03

**Authors:** Ke-Ni Yang, Chun-Qiu Zhang, Wei Wang, Paul C. Wang, Jian-Ping Zhou, Xing-Jie Liang

**Affiliations:** ^1^State Key Laboratory of Natural Medicines, Department of Pharmaceutics, China Pharmaceutical University, Nanjing 210009, China; ^2^CAS Key Laboratory for Biological Effects of Nanomaterials and Nanosafety, National Center for Nanoscience and Technology, Chinese Academy of Sciences, Beijing 100190, China; ^3^Laboratory of Molecular Imaging, Department of Radiology, Howard University, Washington DC 20060, USA

**Keywords:** Mesoporous silica nanoparticles, pH-responsive, controlled drug release, drug delivery systems, antineoplastic protocols

## Abstract

In the fight against cancer, controlled drug delivery systems have emerged to enhance the therapeutic efficacy and safety of anti-cancer drugs. Among these systems, mesoporous silica nanoparticles (MSNs) with a functional surface possess obvious advantages and were thus rapidly developed for cancer treatment. Many stimuli-responsive materials, such as nanoparticles, polymers, and inorganic materials, have been applied as caps and gatekeepers to control drug release from MSNs. This review presents an overview of the recent progress in the production of pH-responsive MSNs based on the pH gradient between normal tissues and the tumor microenvironment. Four main categories of gatekeepers can respond to acidic conditions. These categories will be described in detail.

## Introduction

Cancer is the leading cause of death worldwide. Conventional chemotherapy is often characterized by clinical inefficiency and serious side-effects, mainly because of the leaking out of drugs during blood circulation and nonspecific cell/tissue biodistribution. The development of nanotechnology and nanomedicine in the past decades has facilitated the development of various nanovehicles for experimental and clinical application as drug delivery systems to solve these problems^1^^,^^2^. Nanovehicles benefit from surface properties and nanoscales and can thus accumulate in tumor tissue effectively with grafted multiple targeting ligands for ‘active targeting’, while exhibiting enhanced permeability and retention effect (EPR) for ‘passive targeting’, which mainly improves local drug concentration and reduces nonspecific tissue biodistribution^3^^-^^5^. Nanovehicles can carry a large payload of cargoes and be conveniently modified to perform desirable functions, such as controlling drug release^6^, improving blood circulation half-life^7^, increasing bioavailability, and bypassing multidrug resistance mechanism^8^^,^^9^.

The most commonly used nanovehicles include liposomes^10^, micelles^11^, dendrimers^12^, nanoparticles^13^, and inorganic materials^14^. However, several barriers block clinical translocation of these nanovehicles to a certain extent because of the premature release and early extraction before reaching the target, uncontrollable rate of release to obtain low local concentration, and inefficient cellular uptake and endosomal escape^15^^-^^17^. Thus, controlled drug delivery systems should be designed. In such systems, controlled drug release at special time and space on demand can be achieved with a ‘zero release’ effect in blood circulation to protect healthy tissues from toxic drugs and to prevent drug decomposition. Several controlled drug delivery nanovehicles based on organic platforms have been fabricated^18^^-^^20^. Discoveries based on inorganic materials have recently opened up new and exciting possibilities in designing controlled drug delivery systems. These materials include gold nanoparticles^14^, magnetic nanoparticles^21^, and silica nanoparticles^22^.

**Figure 1:**
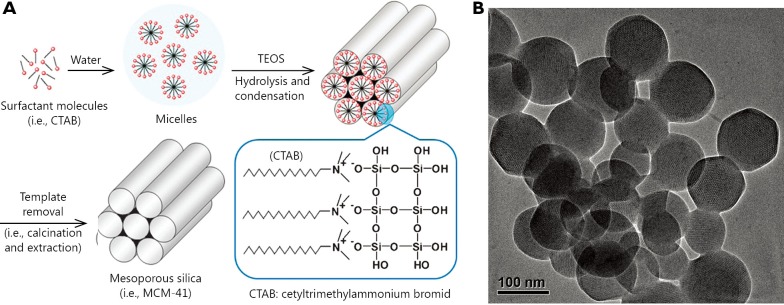
Synthesis scheme for the preparation of MSNs (A) and transmission electron microscopy (TEM) images of MCM-41 (B). (**Figure 1A** is adapted from Ref. 22 with permission of The Royal Society of Chemistry).

Among these inorganic materials, mesoporous silica nanoparticles (MSNs) have aroused significant interest and rapidly developed into an important candidate for nanomedical applications since a MCM-41-type mesoporous silica material was first reported as a drug delivery system in 2001^23^. As shown in **Figure 1**, the simple, scalable, and cost-effective fabrication, as well as non-toxic nature, large surface area and pore volume, and high density silanol-containing surface are apparent advantages of MSNs. On one hand, the textural characteristics of MSNs increase the loading amount of anti-cancer drugs that are encapsulated in pore tunnels. On the other hand, the silanol-containing surface can be easily modified with various molecules, resulting in an enhanced profile for the pharmacokinetics and pharmacodynamics of therapeutic agents^22^. Moreover, the nanotunnels that encapsulate cargoes can be sealed with various gatekeepers, and such cargoes will not be released until triggered by stimuli, which offers an opportunity to design stimuli-responsive drug delivery systems for controlled release.

The stimulus can be divided into endogenous stimulus and exogenous stimulus^17^. Endogenous stimuli arise from the microenvironment differences between normal tissues and tumor, such as reduced intercellular/intracellular pH, higher redox potential, and increased level of certain enzymes^24^^,^^25^. However, exogenous stimuli are based on extracorporeal physical alterations, including temperature changes, magnetic fields, ultrasounds, as well as light and electric fields^17^. Among these stimuli, low pH is easy to achieve and has become the focus of numerous investigations in oncology because the extracellular pH of normal tissues and blood is approximately 7.4, whereas that in a tumor microenvironment is between 6.0 and 7.0, which is mainly caused by high glycolysis rate and high level of CO_2_^26^. The pH value will drop further from the extracellular microenvironment of a tumor to intracellular organelles, such as endosomes (pH=5.5) and lysosomes (pH<5.5). Therefore, the abnormal pH gradients combined with the advantages of MSNs provide opportunities to realize pH-responsive MSNs as controlled drug delivery systems for cancer treatment.

Many groups have reported on pH-responsive MSNs modified with various gatekeepers. The triggered release of anti-cancer drugs from nanotunnels of mesoporous materials has mainly been achieved by using polyelectrolytes, supramolecularnanovalves, pH-sensitive linkers, and acid-decomposable inorganic materials^27^.

In this paper, we review the recent advances in drug delivery of pH-responsive MSNs with four categories of gatekeepers for cancer treatment based on tumor microenvironment.

## pH-responsive MSNs with polyelectrolytes gatekeepers

Polyelectrolytes, which are polymers with repeating units that bear electrolyte groups, are either absorbed or covalently bonded to the surface of MSNs to serve as a mechanized stimulus-responsive release system by transformation under different pH values^28^. Under neutral or weakly basic conditions, the polyelectrolytes tightly wrap around the particle surface and block multiple openings. With decreasing pH value, the polyelectrolytes are triggered to go through swelling or coiling so that the cargoes are released from the unblocked pores^29^.

**Figure 2:**
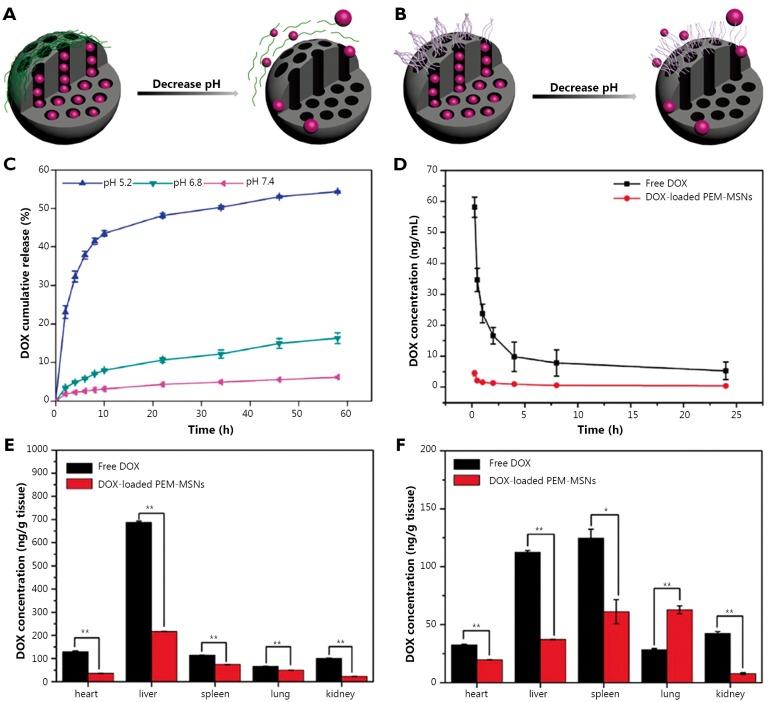
Graphical representation of pH-responsive MSNs with polyelectrolyte multilayers (A) and polyelectrolyte brushes (B). Release profiles of DOX from PEM-MENs (eight layers) in different pH media (C). DOX concentrations in plasma after DOX and DOX-loaded PEM-MSNs were injected intravenously through the vein for incremental time (D). Biodistribution of DOX in healthy SD rats at 2 h (E) and 24 h (F) after DOX and DOX-loaded PEM-MSNs at 2 mg/kg DOX equivalent were injected intravenously through the vein. *P<0.05 and **P<0.01 compared with free DOX group. (**Figure 2C,D,E,F** are adapted from Ref. 30 with permission from The Royal Society of Chemistry).

Feng *et al*.^30^ synthesized a type of pH-responsive MSNs with polyelectrolyte multilayers (PEM) composed of poly (allylamine hydrochloride) (PAH) and sodium poly(styrene sulfonata) (PSS) using a layer-by-layer technique. A schematic illustration of the construction and release mechanism of PEM-MSNs is shown in **Figure 2A**** and ****2B****.** The PEM-MSNs with eight polymer layers possess maximum encapsulation efficiency, and the release of DOX is accelerated under acidic conditions with incompact PAH/PSS multilayers (**Figure 2C**). In HeLa cells, DOX-loaded PEM-MSNs are almost distributed in the cytoplasm within 6 h, and some DOX is released from carriers into the nucleus for 12 h. Meanwhile, free DOX rapidly enter cancer cells and accumulate in the nucleus within 0.5 h. Thus, DOX-loaded PEM-MSNs are internalized into endosomes/lysosomes, and then pH-triggered DOX release occurs from nanotunnels because of the low pH (~5.0) in the endosomes/lysosomes followed by the delivery of released DOX from cytoplasm to nucleus. This process prolongs the accumulation of DOX in the nucleus to enhance the anti-cancer efficiency. Moreover, the blood profiles of DOX after intravenous injection of free DOX and DOX-loaded MSNs show different patterns (**Figure 2D**). The majority of free DOX have a rapid clearance within 2 h of administration, followed by a slow clearance phase. By contrast, DOX-loaded PEM-MSNs show low and sustained drug concentration in rat plasma up to 24 h post-injection, possibly because of the relatively high pH value of blood and the close state of PEM-MSNs. Furthermore, the determination of DOX levels in major organs, as well as the histological examination, indicates that DOX-loaded PEM-MSNs have lower systemic toxicity than free DOX (**Figure 2E,F**).

Sun *et al*.^31^ selected poly[2-(diethylamino)ethyl methacrylate] (PDEAEMA) to functionalize the MSNs through the surface-initiated atom transfer radical polymerization of DEAEMA. Under neutral and alkaline conditions, PDMAEMA chains are prone to aggregate together with polymer chain-chain interactions to seal the nanotunnels of MSNs. However, under acid conditions, the tertiary amine in PDEAEMA can easily obtain a proton to form quaternary ammonium. This process is followed by polymer chain stretching with the electrostatic repulsions and strong chain-solvent interaction (**Figure 2B**), which facilitates cargo release. Yang *et al*.^32^^-^^36^ also studied the ways by which to employ polyelectrolytes as pH-responsive gatekeepers. For instance, in their pH-sensitive system of poly (glutamic acid) grafted MSNs (MSN-PLGA), the drug loading experiment was performed at pH 8.0 because of the electrostatic attraction between DOX and the nanoparticles. The drug release behavior of MSN-PLGA loaded DOX was then studied at different pH values (5.5, 6.8, and 7.4). The results indicated that MSN-PLGA had high drug loading efficiency and exhibited a significantly pH-dependent drug release behavior. This finding can be attributed to the fact that the protonation of poly (glutamic acid) with decreasing pH results in the dissociation of the electrostatic interaction between PLGA and DOX and consequently facilitates DOX release. Many other polyelectrolytes were developed as gatekeepers for designing pH-responsive MSNs, such as poly(4-vinyl pyridine)^37^, chitosan^38^^,^^39^ and poly(acrylic acid)^40^. Thus, the weak acid tumor tissues make pH-responsive release systems suitable for the controlled release of anti-cancer drugs.

## pH-responsive MSNs with supramolecularnanovalves

**Figure 3:**
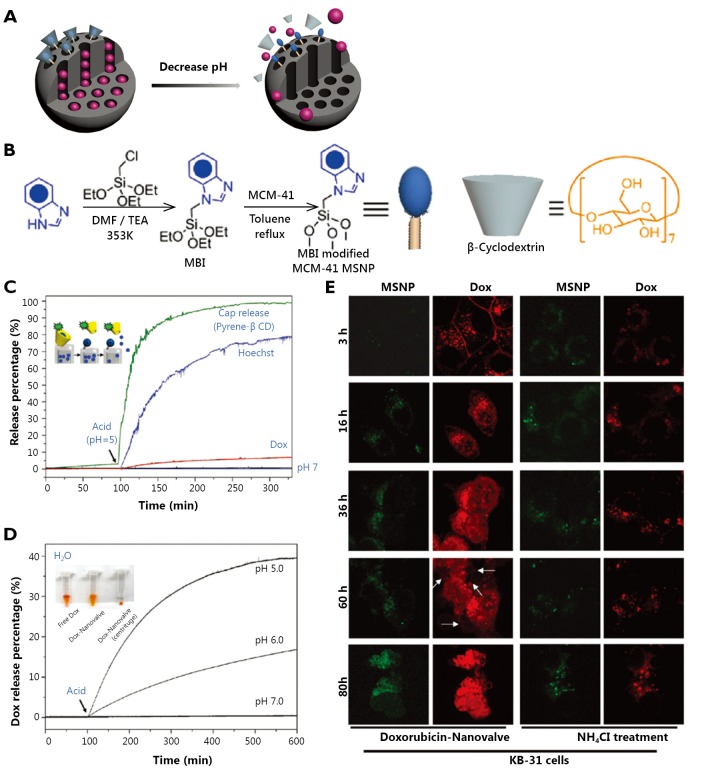
Graphical representation of the pH-responsive MSNs with supramolecularnanovalves (A). Synthesis of the stalk on the surface of MSNs for further β-CD capping on the pore (B). Fluorescence intensity plots for the release of Hoechst dye, doxorubicin, and the pyrene-loaded cyclodextrin cap from MSNs (C) and release profiles of doxorubicin from ammonium-modified (7.5%, w/w) nanoparticles showing a faster and larger response compared with that of unmodified MSNs (D). Confocal images of KB-31 cells incubated with MSNs containing doxorubicin for the indicated times: KB-31 cancer cells effectively endocytosed the doxorubicin-loaded FITC-MSNs at 3 h. This action is followed by nuclear fragmentation after 80 h. However, with NH4Cl treatment, most of the doxorubicin was confined to nanoparticles, such that no observable cell death occurred (E). (**Figure 3B,C,D,E** are adapted with permission from Ref. 42. Copyright 2010, American Chemical Society).

The development of supramolecular chemistry enabled supramolecular assembly to be made into a ‘nanovalve’ machine responding to various stimuli, such as chemical, light, and electrical stimuli^41^. The supramolecularnanovalve, as a gatekeeper for controlling cargo release, includes an immobilized stalk molecule covalently attached to silica surface and a mobile cyclic molecule encircling the stalk via non-covalent interactions^29^. Under certain conditions, the binding constant between cyclic caps and stalks weakens, thus resulting in large-amplitude sliding motions of the caps and the unblocking of nanotunnels. Therefore, supramolecularnanovalves provide opportunities for MSNs to construct a pH-responsive drug delivery system for responding to weak acidic tumor tissues (**Figure 3A**).

Meng *et al*.^42^ reported a novel MSNs delivery system based on the function of *β*-cyclodextrin (*β*-CD) nanovalves that were responsive to the acidic conditions of endosomes in cancer cells. In this system, *N*-methylbenzimidazole is chosen to serve as stalks for the optimized pK_a_ (5.67) (**Figure 3B**), which binds to the *β*-CD rings strongly at pH 7.4 with trapping cargoes in nanotunnels while causing dissociation with the *β*-CD caps at pH <6 in the acidifying endosomal compartment. The profiles of drug release accompanied by *β*-CD detachment from MSNs before and after acid stimuli are presented in **Figure 3C**, which shows typical pH-responsive release characteristics. To improve the rate and quantity of DOX release, the interior of the silica surface is modified with 7.5% ammonium (**Figure 3D**). In squamous carcinoma (KB-31) cells, DOX-loaded nanoparticles are taken up into the perinuclear regions efficiently within 3 h, where drug release to the nucleus is observed. The release is followed by apoptosis at 60 h, nuclear fragmentation after 80 h, and finally cell death (**Figure 3E**, left panel). However, the release profile of DOX dramatically changes after NH_4_Cl treatment of KB-31 cells, in which most drugs are retained inside nanotunnels and little evidence of nuclear staining and cell death is observed (**Figure 3E**, right panel). In addition, quantitative analysis of the nuclear DOX fluorescence signal and MTS assays further confirm that the cargo release caused by lysosomal acidification is made feasible by the pH-sensitive nanovalves during *in vitro* operation.

Similarly, Du *et al*.^43^ successfully synthesized a biocompatible pH-responsive nanovalve based on MSNs comprising α-cyclodextrin (α-CD) rings and *p*-anisidino linkers modified on the silica surface. Luminescence spectroscopy demonstrates that the pH-responsive system exhibits good bio-stability and no drug leakage at pH ~7.4, as well as excellent drug release performance not only in H_2_O but also in cell culture medium at pH ~5.5 upon the protonation of *p*-anisidino nitrogen atoms (part of the linker). Therefore, Du *et al*. explored the applications of the α-CD nanovalves based on MSNs by testing their delivery capability in different types of human cancer cells at lysosomal pH levels.

In addition, cucurbit[n]uril, the structure of which is similar to cyclodextrin, is capable of blocking the pores of MSNs as nanovalves and of preventing the cargoes from leaking out until they are detached from the stalks or positioned far away from the pore entrances by sliding under acidic stimuli^41^. In a typical design, Angelos *et al*.^44^ developed supramolecularnanovalves composed of cucurbit(6)uril [CB(6)]/trisammoniumpseudorotaxanes that are attached to MSNs surfaces and encapsulate cargo molecules at neutral pH and then release the cargoes under mildly acidic conditions. Owing to the difference in the binding affinity of CB(6) with NH_3_^+^(CH_2_)_6_NH_3_^+^ and NH_3_^+^(CH_2_)_4_NH_3_^+^, the CB(6) ring shuttles to the distal hexamethylenediammonium recognition unit once the anilinium nitrogen atom protonated, which then unblocks the pore orifice and facilitates cargo release. More importantly, the pH at which the MSNs system responds can be tuned through rational chemical modification of the stalk.

## pH-responsive MSNs with pH-sensitive linkers

**Figure 4:**
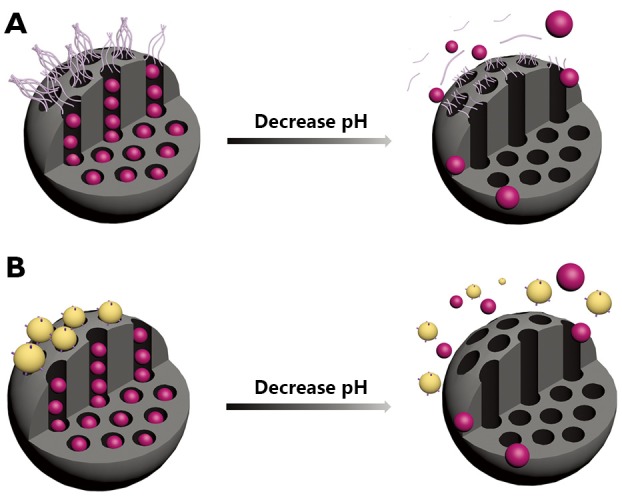
Graphical representation of the pH-responsive MSNs capped with polymers (A) and nanoparticles (B) that linked to the surface of MSNs via pH-sensitive linkers.

The pH-sensitive linkers, such as acetal bond, hydrazine bond, and ester bond can be cleaved with decreasing pH value, thus providing opportunities for designing pH-responsive MSNs. On one hand, the pH-sensitive linkers modified over the pore entrances of MSNs can induce bulky groups as nanogates to block the pores and control drug release (**Figure 4**). On the other hand, the pH-sensitive linkers can also be modified in the nanotunnels to bond with drugs covalently. These drugs will then be released with the cleavage effects of linkers between drugs and MSNs under acidic conditions.

Gao *et al*.^45^ employed functionalized MSNs as drug reservoirs and then blocked the mesopores with polypseudorotaxanes through pH-sensitive benzoic-imine bonds hydrolyzed under very weak acidic conditions but stable at neutral basic pH because of the proper π-π conjugation extent. The polypseudorotaxanes consist of poly (ethylene glycol) (PEG) and α-CD, with PEG serving as the guest polymer for CD hosts and imparting *in vivo* longevity to MSNs by preventing nonspecific protein adsorptions during the circulation. Under weak acidic tumor extracellular pH (~6.8), the benzoic-imine linkages begin partially hydrolyzing to accelerate DOX release and meanwhile generate positive amino groups to facilitate internalization of particles. Subsequently, in the more acidic endosomal pH (~5.0), the increasing hydrolysis of the benzoic-imine bond would intensify the removal of the polypseudorotaxane caps and thus accelerate the release of DOX into the cytoplasm. In HepG2 cells, DOX-loaded MSNs are initially located within endosomal intracellular compartments and release drugs in the cytosol region in a sustained manner. Moreover, the different results of confocal fluorescence microscopy and cytotoxicity assay between cells exposed to DOX-loaded MSNs at pH =6.8 and pH =7.4 again prove that enhanced tumor-specific uptake and intracellular delivery can be achieved through the inclusion of the benzoic-imine linkage.

Analogously, Liu *et al*.^46^ reported a new pH-responsive nanogated construction by capping gold nanoparticles onto mesoporous silica through acid-labile acetal linkers (**Figure 4B**). At neutral pH, the linker remains intact, and pores are blocked by gold nanoparticles to inhibit cargo diffusion. However, at acidic pH, the hydrolysis of the acetal group removes the caps and allows release of cargoes. Aside from bulky groups capped on the outlets via pH-sensitive linkers, Lee *et al*.^47^ conjugated DOX to the inner wall of MSNs nanochannels via liable hydrazone bonds. Through EPR effects, the Atto-647-MSN-hydrazone-DOX inherently accumulates in solid tumors of the liver. Nanoparticles then highly concentrate within endosomes and lysosomes of cancer cells. Sustained release of drug payload is observed because of the leakage of hydrazone bonds at endosomal and lysosomal pH. Moreover, apart from DOX, the pH-sensitive drug release mechanism can be applied to other anti-cancer drugs that possess functional ketones or aldehydes.

## pH-responsive MSNs with acid-decomposable inorganic gatekeepers

**Figure 5:**
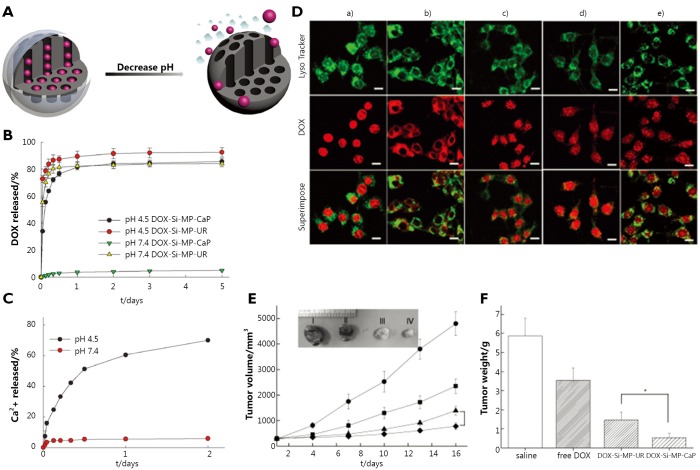
(A) Graphical representation of pH-responsive MSNs with acid-decomposable inorganic gatekeepers. (B) DOX release profiles from DOX-Si-MP-UR and DOX-Si-MP-CaP under pH control. (C) Kinetics of calcium dissolution from DOX-Si-MP-CaP under pH control. (D) CLSM images of live MCF-7 cells treated with Lyso Tracker (50 nm), free DOX (5 µg/mL), and DOX-Si-MP-CaP (DOX =5 µg/mL), thereinto, (a) free DOX for 1 h exposure; (b) DOX-Si-MP-CaP for 1 h exposure; (c) DOX-Si-MP-CaP for 5 h exposure; (d) DOX-Si-MP-UR for 1 h exposure; and (e) DOX-Si-MP-UR for 5 h exposure. (Green fluorescence is associated with Lyso Tracker; the red fluorescence is expressed by free DOX, released DOX, and DOX retained within MSNs). Scale bar: 20 µm. (E) *In vivo* therapeutic efficacy after a single intratumoral injection of saline (●), free DOX (█), DOX-Si-MP-UR (▲), and DOX-Si-MP-CaP(◆) at a DOX-equivalent dose of 10 mg/kg. Inset: images of excised tumors at 16 days after treatment. I: saline, II: free DOX, III: DOX-Si-MP-UR, IV: DOX-Si-MP-CaP. (F) Tumor weights at 16 days after treatment. The results represent the means ± SDs (*n*=4); *P<0.05. (**Figure 4B,C,D,E,F** are adapted from Ref. 48 with permission of John Wiley and Sons).

The common strategies used for surface functionalization include grafting organic species. However, such strategies have been limited by tedious and intricate organic synthesis steps and the lack of a clear definition of the body toxicity of dismantled pore-blocking agents. Some acidic-decomposable inorganic materials have recently been reported as gatekeepers to control drug release, offering opportunities to design promising specific carriers for therapeutic agents (**Figure 5A**).

Rim *et al*.^48^ introduced inorganic calcium phosphate (CaP) as a novel pore blocker through the enzyme-mediated mineralization on the MSN surface, which can be dissolved in intracellular endosomes as nontoxic ions to initiate drug release. The construction of the nanoparticle involves urease functionalization of MSN surfaces and subsequent enzyme-mediated surface CaP mineralization in the presence of urea under mild conditions within a short time. For pH-controlled DOX release from mineralized MSNs, pH variation between physiological pH (pH 7.4) and low pH (pH 4.5) is employed. The results show that a large amount of DOX was released after 24 h under low pH conditions (**Figure 5B**). Furthermore, the pH-dependent dissolution kinetics of Hap-like coating support the DOX release profiles from CaP capped MSNs (**Figure 5C**), which confirms that the dissolution of pore blocks results in the opening of the pore and then triggers DOX release. In breast cancer MCF-7 cells, DOX-loaded mineralized MSNs (DOX-Si-MP-CaP) carry DOX in nanopores effectively before endocytosis, and DOX release can be facilitated in lysosomes by the dissolution of mineral coatings, followed by the DOX release and accumulation in the nucleus (**Figure 5D**). Moreover, the evaluation of the *in vivo* efficacy of DOX-Si-MP-CaP using xenograft models of MCF-7 human breast cancer shows that a single intratumoral administration of DOX-Si-MP-CaP is significantly more effective in tumor reduction than control groups including free DOX and DOX-Si-MP-CaP (**Figure 5E,F**).

Muhammad *et al*.^49^ employed acid-decomposable luminescent ZnO quantum dots (QDs) to seal the nanopores of MSNs in order to inhibit premature drug (DOX) release. After internalization into HeLa cells, the ZnO QD lids are dissolved rapidly in the acidic intracellular compartments, followed by loaded drug release from MSNs into cytosol. In this pH-responsive drug delivery system, ZnO QDs behave as a dual-purpose entity that not only serves as a lid but also imposes a synergistic anti-tumor effect on cancer cells. Zheng *et al*.^50^ reported a pH-responsive controlled release system via using acid-decomposable layered double hydroxides (LDHs) as inorganic nanovalves, by virtue of the electrostatic interaction of LDH nanosheets on the surface of MSNs. The preparation procedure of the pH-responsive MSNs is free from complicated organic synthesis. Guest molecules are loaded and capsulated in neutral and released in acidic pH depending on the dissolution of LDHs. Thus, acid-decomposable inorganic materials are promising candidates for designing pH-responsive MSNs.

## Conclusion and outlook

In this review, we highlighted the exciting research advances on pH-responsive drug delivery systems based on MSNs. Various materials can be used as gatekeepers to control drug release under acidic conditions. These materials have great potential for application in tumor therapy and for improving anti-cancer drug efficiency and decreasing side effects. However, most work is focused on *in vitro* studies^51^. Thus, several challenges still need to be overcomed for the further advancement of the biological and biomedical applications of pH-responsive MSNs. First, the differences in the pH values between tumor microenvironment and normal tissues are minimal, making the manipulation of the stimuli-responsive drug delivery system *in vivo* via pH difficult. Second, the targeting effects of pH-responsive MSNs depending on EPR effects are low, thus causing nanoparticles accumulation in some organs, such as the heart, liver, and spleen. Upon accumulation in normal tissues, MSNs can be internalized into cells via endocytosis to trigger drug release, which may result in side effects. Third, the biodistribution, acute and chronic toxicities, changes in molecule level, long-term stability, and circulation properties of stimulus-responsive drug delivery systems need to be further investigated before implementation in clinical practice. Therefore, future work in designing stimulus-responsive MSNs will most likely be directed toward the integration of multiple stimuli strategies that can respond to two or more stimuli simultaneously and can bear targeting molecules for efficiently directing the nanoparticles to tumor tissues, with low toxicity and good pharmacokinetic profile.
